# The protective role of resilience-related strength in the association between sleep reactivity and insomnia symptoms among young Chinese male university students: a moderated network approach

**DOI:** 10.3389/fpsyt.2026.1905193

**Published:** 2026-08-27

**Authors:** Donghe Li, Dangyang Ma, Tao Chen, Hui-feng Duan, Xue-jun Liang, Zhansheng Xu

**Affiliations:** 1 Mental Diseases Prevention and Treatment Institute of Chinese PLA, No. 988 Hospital of Joint Logistics Support Force, Jiaozuo, China; 2 Faculty of Health and Wellness, City University of Macau, Macao, Macao SAR, China; 3 Faculty of Psychology, Tianjin Normal University, Tianjin, China; 4 Key Research Base of Humanities and Social Sciences of the Ministry of Education, Academy of Psychology and Behavior, Tianjin Normal University, Tianjin, China; 5 Center of Cooperative Innovation for Assessment and Promotion of National Mental Health under the Ministry of Education, Tianjin Normal University, Tianjin, China

**Keywords:** insomnia symptoms, moderated network model, multiple regression, psychological resilience, sleep reactivity

## Abstract

**Background:**

Sleep reactivity is closely associated with insomnia symptoms, but whether this association is moderated by different dimensions of psychological resilience remains insufficiently understood. This study examined the associations among sleep reactivity, psychological resilience, and insomnia symptoms in a sample of young Chinese male university students.

**Methods:**

A total of 1,122 male participants (*Mean_a_*_ge_ = 21.13, *SD*_age_ = 1.48) were included in the analyses. Sleep reactivity, insomnia symptoms, and psychological resilience were assessed using the Ford Insomnia Response to Stress Test, the Insomnia Severity Index, and the Connor-Davidson Resilience Scale, respectively. Multiple regression was first used to compare the main and moderating effects of three resilience dimensions: Tenacity, Strength, and Optimism. A moderated network model was then estimated to localize moderation effects at the level of specific sleep reactivity-insomnia links.

**Results:**

Multiple regression showed that sleep reactivity was positively associated with insomnia symptoms. Among the three resilience dimensions, only Strength was negatively associated with insomnia symptoms and significantly moderated the association between sleep reactivity and insomnia symptoms under conventional OLS inference. Moderated network analysis further showed that Strength moderated specific links between sleep reactivity components and insomnia symptoms, particularly the links involving sleep reactivity related to interpersonal conflict and public speaking.

**Conclusion:**

Strength may be an important protective psychological resource that weakens the association between stress-related sleep vulnerability and insomnia symptoms in men. These findings suggest preliminary evidence that post-stress recovery capacity may be relevant to understanding individual differences in the association between sleep reactivity and insomnia symptoms among young men.

## 1 Introduction

Insomnia is one of the most common sleep problems and is typically characterized by difficulty initiating sleep, difficulty maintaining sleep, early-morning awakening, or non-restorative sleep, accompanied by varying degrees of daytime impairment, such as fatigue, reduced attention, emotional distress, and poorer quality of life (1, 2). Its onset, persistence, and chronicity can be understood within the three-factor model of predisposing, precipitating, and perpetuating factors. According to this model, individual-level predisposing factors increase the baseline risk of developing sleep problems. On this basis, precipitating factors, such as stressful events, life changes, or emotional distress, may trigger acute sleep disturbance. If maladaptive sleep-related cognitions, compensatory behaviors, and persistent physiological arousal (3, 4) subsequently emerge as perpetuating factors, insomnia symptoms may persist even after the initial precipitating factors have weakened and may gradually develop into chronic insomnia (5–7). Therefore, this model not only highlights the role of external precipitating factors in the onset of insomnia but also suggests that individuals differ in their sleep responses to similar precipitating contexts.

Sleep reactivity is an important construct that captures this individual difference. It refers to a stable vulnerability of the sleep system to become disturbed under conditions of stress, challenge, or emotional arousal (8–14). Individuals with high sleep reactivity are more likely to experience difficulty initiating sleep, difficulty maintaining sleep, or reduced sleep quality when facing similar precipitating factors. Therefore, sleep reactivity is considered a key vulnerability factor in the onset and chronicity of insomnia. Unlike indicators of current insomnia severity, sleep reactivity emphasizes the tendency to develop sleep disturbance under stressful conditions and is therefore conceptually closer to a predisposing risk trait in the 3P model. Longitudinal evidence further supports the theoretical status of sleep reactivity as an insomnia vulnerability: baseline sleep reactivity predicts the subsequent onset of insomnia, and this predictive effect remains evident even among individuals without a prior history of insomnia or depression (15–17). These findings suggest that sleep reactivity is not merely a concomitant manifestation of current insomnia symptoms but may represent an antecedent risk trait that exists before insomnia symptoms are fully developed.

Psychological resilience has been considered a key psychological resource that may buffer the impact of stress-related sleep reactivity on insomnia. Connor and Davidson (18) defined resilience as the capacity to cope with stress and adversity, which helps individuals maintain or regain adaptive functioning under unfavorable conditions. In Chinese populations, psychological resilience can be further divided into three interrelated but conceptually distinct dimensions: Tenacity, Strength, and Optimism. Tenacity emphasizes persistence, composure, and a sense of control when facing challenges; Strength focuses on recovery, growth, and self-enhancement after setbacks; and Optimism reflects the tendency to view adversity positively and to trust personal or social resources (19). This multidimensional structure suggests that the protective effect of resilience against insomnia vulnerability may not arise from a single global resilience level but may instead reflect differentiated roles of specific resilience components.

Previous sleep-related studies have provided preliminary evidence for associations between psychological resilience and sleep-related outcomes. A systematic review and meta-analysis showed that sleep quality and sleep duration were generally positively associated with overall resilience, although the effect sizes were small and more prospective studies are needed to clarify the temporal and causal directions of these associations (20). Although most previous studies have treated resilience as an overall construct, emerging evidence suggests that its dimensions may not be interchangeable in relation to insomnia. A recent network study using the Chinese three-factor CD-RISC found that Tenacity, Strength, and Optimism were all negatively associated with insomnia symptoms but occupied different positions within the broader psychosocial network (21). This finding suggests that different resilience dimensions may capture partly different aspects of the relationship between resilience and sleep and therefore supports examining their associations with insomnia severity separately. Importantly, the relationship between sleep and psychological resilience may also be reciprocal. Resilience may support sleep through adaptive stress appraisal, emotion regulation, and post-stress recovery, whereas persistent sleep disturbance may impair the emotional and cognitive resources required for adaptive coping, thereby contributing to lower perceived resilience (20, 22).

Sleep reactivity and psychological resilience may also be closely interrelated. In a sample of individuals with insomnia disorder, Palagini et al. (23) found that participants with insomnia showed lower psychological resilience and higher stress-related sleep reactivity. After controlling for anxiety and depressive symptoms, lower resilience remained associated with greater stress-related sleep reactivity, cognitive presleep arousal, and difficulties in emotion regulation. Rather than indicating a single directional risk pathway, these findings point to close interrelations among psychological resilience, sleep reactivity, presleep arousal, and insomnia. Therefore, examining resilience within the framework of sleep reactivity may help clarify whether the association between sleep reactivity and insomnia severity varies across resilience dimensions.

The above vulnerability-protection mechanism may also be influenced by gender. Existing evidence indicates stable gender differences in insomnia, with women generally showing a higher prevalence of insomnia than men (24). However, gender differences are not limited to prevalence; they may also be reflected in precipitating factors, clinical manifestations, comorbidity patterns, and sleep-circadian regulatory mechanisms (25, 26). In addition, the genetic and environmental basis of sleep reactivity may also differ by gender (27). Therefore, examining the associations among sleep reactivity, psychological resilience, and insomnia severity in a male sample may help reveal vulnerability and protective mechanisms within men while relatively reducing heterogeneity related to gender.

Although previous studies have provided an important basis for understanding the associations among sleep reactivity, psychological resilience, and insomnia, two gaps remain. First, existing studies have mostly discussed the protective role of resilience using the overall level of resilience or a single resilience dimension. Few studies have simultaneously compared the independent contributions of Tenacity, Strength, and Optimism to the association between sleep reactivity and insomnia. Therefore, it remains unclear which resilience component is most likely to play the primary buffering role after controlling for the shared variance among the three dimensions, and whether the dimensions exhibit distinct moderating patterns. To address this gap, multiple regression allows the three resilience dimensions to be entered into the same model, thereby comparing their relatively independent main effects and interaction effects.

Second, traditional variable-centered approaches can estimate interaction effects at the global level but cannot determine whether such patterns are concentrated in particular stress-related contexts. Network analysis has increasingly been used in sleep and mental health research to characterize conditional associations among multiple variables while accounting for the remaining variables in the system (28, 29). The moderated network model (MNM) extends this approach by examining whether the conditional association between two nodes varies as a function of a moderator (30), thereby providing a context-level extension to conventional scale-level moderation analysis.

Accordingly, the present study examined the associations among sleep reactivity, psychological resilience, and insomnia severity in a sample of young Chinese male university students using a sequential analytic strategy. Joint multiple regression was first used to compare the main and interaction effects of Tenacity, Strength, and Optimism at the scale level. Any resilience dimension showing a candidate interaction with sleep reactivity was then carried forward as the moderator in a subsequent exploratory MNM. This sequential strategy combined a comparison of resilience dimensions with an examination of potentially context-specific moderation patterns.

## 2 Methods

### 2.1 Participants

The current study used convenience sampling to recruit male participants from local universities in Jiaozuo, Henan Province, China, between 2024 and 2025. Data were collected using offline paper-and-pencil questionnaires. A total of 1,192 participants were initially approached. After excluding 70 questionnaires with missing data on one or more study scales, 1,122 valid questionnaires remained, yielding a valid-response rate of 94.1% (1,122/1,192). All participants provided written informed consent. The study protocol was reviewed and approved by the Committee on Ethics of Medicine, The 988th Hospital (approval number: 988YY20240002LLSP).

Participants ranged in age from 17 to 29 years, with a mean age of 21.13 years (SD = 1.48). A total of 198 participants (17.6%) had completed high school or vocational education or below, whereas 924 (82.4%) had received university education or above. Smoking was reported by 408 participants (36.4%), and alcohol use was reported by 178 participants (15.9%).

### 2.2 Measures

#### 2.2.1 Ford insomnia response to stress test

The Ford Insomnia Response to Stress Test (FIRST; 8) was used to assess sleep reactivity, defined as the tendency to experience sleep disturbance in response to stressful situations (10, 13). The scale consists of 9 items describing different stress-related scenarios in which individuals may have difficulty initiating or maintaining sleep. Each item is rated on a Likert-type scale, and the total score ranges from 9 to 36; higher scores indicate a greater likelihood of sleep disturbance and heightened arousal under stress. In the current study, the FIRST showed good internal consistency, with a Cronbach’s alpha of 0.896.

#### 2.2.2 Insomnia severity index

The Insomnia Severity Index (ISI; 31–33) was used to assess the severity of insomnia symptoms and related daytime impairment. The scale consists of 7 items assessing sleep-onset difficulties, sleep-maintenance difficulties, early-morning awakening, satisfaction with current sleep patterns, interference with daily functioning, noticeability of impairment attributed to sleep problems, and distress or worry caused by sleep difficulties. Each item is scored from 0 to 4, and the total score ranges from 0 to 28. Higher scores indicate more severe insomnia symptoms. In the current study, the ISI showed good internal consistency, with a Cronbach’s alpha of 0.909.

#### 2.2.3 Connor-Davidson resilience scale

The Connor-Davidson Resilience Scale (CD-RISC; 18) was used to assess psychological resilience. The scale consists of 25 items rated from 0 to 4, with higher scores indicating greater resilience. The Chinese version comprises three dimensions: Tenacity, Strength, and Optimism (19). Tenacity reflects persistence, determination, and perceived control when facing adversity; Strength reflects the capacity to recover from setbacks and adapt to stressful situations; and Optimism reflects a positive attitude toward change and confidence in coping with future challenges. In the present sample, internal consistency was good for the overall CD-RISC (α = .937), Tenacity (α = .895), and Strength (α = .873), but was low for Optimism (α = .509). The three subscale scores were strongly and positively correlated. The strongest correlation was observed between Tenacity and Strength (r = .853, p <.001), followed by Strength and Optimism (r = .673, p <.001) and Tenacity and Optimism (r = .645, p <.001). An ordinal three-factor confirmatory factor analysis using the weighted least squares mean- and variance-adjusted estimator was additionally conducted to examine whether the three dimensions were empirically distinguishable in the present sample.

### 2.3 Analytic strategy

All statistical analyses were conducted in R 4.5.1 (34). To examine the role of resilience in the association between sleep reactivity and insomnia, we adopted a two-step analytic strategy. First, multiple regression analyses were conducted to identify which resilience dimension showed a potential moderating effect on the overall association between sleep reactivity and insomnia. Second, the identified resilience dimension was incorporated into a moderated network model (MNM; 30) to examine whether and how item-level associations between sleep reactivity and insomnia symptoms varied as a function of the moderator. Specifically, the MNM allowed us to identify which connections between components of sleep reactivity and insomnia were moderated by resilience. Finally, node centrality, network stability, and significant moderation effects were examined.

#### 2.3.1 Multiple regression analysis

In this analysis, the ISI total score was entered as the dependent variable. The FIRST total score, the three CD-RISC subscale scores (Tenacity, Strength, and Optimism), and the interaction terms between FIRST and each resilience dimension were entered simultaneously as predictors (35). All continuous variables, including the dependent variable, were standardized as z scores before the interaction terms were calculated. A resilience dimension showing nominal evidence of moderation (p <.05) in the joint ordinary least squares (OLS) model was carried forward as the moderator in the subsequent exploratory moderated network analysis. Model performance was evaluated using R^2^, adjusted R^2^, and the change in explained variance associated with the interaction terms, while multicollinearity was assessed using variance inflation factors (VIFs) and tolerance values. Detailed procedures for the regression sensitivity analyses, including robustness checks and covariate adjustment for potential demographic and behavioral confounding, are provided in the Supplementary Methods; complete results are reported in **Supplementary Tables 4 and S5**.

#### 2.3.2 Moderated network estimation

To examine whether the candidate scale-level moderation pattern could be localized to specific components of sleep reactivity, an exploratory cross-sectional Gaussian moderated network model was estimated using the modnets package in R (30, 36). The network included the nine FIRST items as separate nodes, the mean of the seven ISI items as a composite insomnia-severity node, and the resilience dimension selected in the preceding regression analysis as the moderator. The seven ISI items were averaged because the instrument was developed and conventionally interpreted as a global index of perceived insomnia severity (31, 33), consistent with our focus on associations between specific sleep-reactivity contexts and the overall construct rather than its internal symptom structure. Moreover, as a linear rescaling of the conventional total score, the ISI mean preserves the same information while limiting model complexity. Accordingly, this model was not intended to characterize associations involving individual insomnia symptoms.

Model selection was performed using hierarchical LASSO, with the optimal regularization parameter selected according to the Bayesian information criterion (BIC). Variables were mean-centered but not scaled, and edges were retained using a nominal threshold of p <.05 and the OR rule, whereby an edge was retained when it was identified in at least one of the corresponding nodewise regressions. The OR rule was selected to prioritize sensitivity in this exploratory localization analysis and to avoid discarding potentially informative edges when statistical support was present in only one of the two corresponding nodewise regressions. Accordingly, retained moderation edges were interpreted as exploratory patterns rather than confirmatory findings. Additional details of moderated-network estimation and stability assessment are provided in the **Supplementary Methods**.

For retained moderation effects, conditional edge weights were examined at low, mean, and high levels of the moderator, defined as one standard deviation below the mean, the mean, and one standard deviation above the mean, respectively. Simple-slope and Johnson–Neyman analyses were used, where applicable, to characterize how these conditional associations varied across levels of the moderator (35, 37).

#### 2.3.3 Centrality and stability assessment

Node centrality was evaluated using expected influence, defined as the signed sum of all edge weights connected to a node (38). The stability of pairwise edge weights and expected influence was assessed using a case-dropping bootstrap with 1,000 resamples. Correlation-stability coefficients above.25 were considered acceptable, whereas coefficients above.50 indicated good stability (28).

## 3 Results

### 3.1 Descriptive statistics and CD-RISC measurement properties

Mean scores for the nine FIRST items ranged from 1.23 to 1.47, suggesting generally low levels of sleep reactivity. Mean ISI total score was 2.70 (SD = 3.81; median = 1; range = 0–28), with marked positive skewness (2.14) and excess kurtosis (6.24). A total of 468 participants (41.7%) scored zero. Based on conventional ISI categories, 997 participants (88.9%) were in the minimal range, 112 (10.0%) in the subthreshold range, 8 (0.7%) in the moderate range, and 5 (0.4%) in the severe range. In contrast, the three resilience dimensions showed relatively higher scores, with Strength (M = 3.33, SD = 0.62) scoring highest, followed by Tenacity (M = 2.99, SD = 0.67) and Optimism (M = 2.52, SD = 0.66). All nine FIRST items were positively skewed (skewness range = 1.37–2.86), whereas Tenacity, Strength, and Optimism showed negative skewness (−0.69, −1.07, and −0.49, respectively).

The ordinal three-factor CFA yielded CFI = .948, TLI = .942, RMSEA = .086 (90% CI [.083,.089]), and SRMR = .055. However, the solution was not fully admissible because the maximum absolute latent correlation was 1.033. Thus, the present data did not provide clear evidence that the three dimensions were empirically distinct latent constructs.

### 3.2 Multiple regression analyses

**Figure 1:**
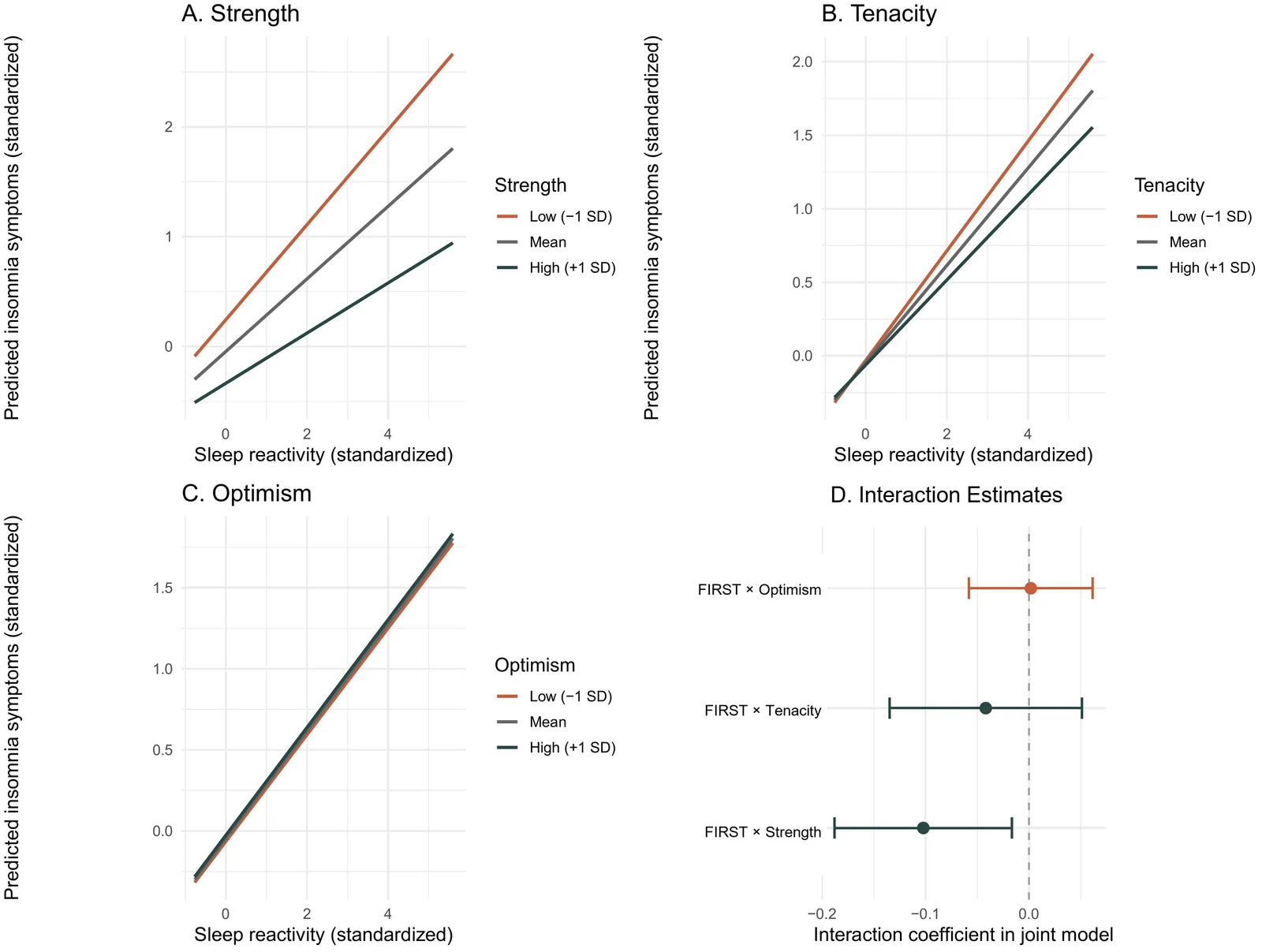
Moderation effects of strength, tenacity, and optimism on the association between sleep reactivity and insomnia symptoms. Panels **(A–C)** display the simple-slope plots, and panel **(D)** shows the interaction estimates from the joint model. error bars in panel **(D)** represent 95% confidence intervals.

The results of the joint multiple regression model are presented in **Table 1**, and the corresponding moderation patterns are shown in **Figure 1**. Sleep reactivity was positively associated with insomnia symptoms (β = 0.332, p <.001). Among the three resilience dimensions, only Strength showed a significant negative main effect (β = −0.279, p <.001), whereas the main effects of Tenacity (β = −0.026, p = .586) and Optimism (β = 0.019, p = .567) were not significant. With respect to moderation, only the FIRST × Strength interaction was significant under conventional OLS inference (β = −0.101, p = .020). As shown in **Figure 1A**, the positive association between sleep reactivity and insomnia symptoms was strongest at low Strength (−1 SD) and weakest at high Strength (+1 SD). The interactions involving Tenacity (β = −0.045, p = .335) and Optimism (β = 0.001, p = .969) were not significant, consistent with the relatively parallel lines in **Figures 1B, C**.

**Table 1:** Joint multiple regression model predicting insomnia symptoms from sleep reactivity, resilience dimensions, and their interaction terms (N = 1,122).

Predictor	*β*	*SE*	*t*	*p*	*95% CI*
Intercept	−.047	.025	−1.87	.062	[−.096,.002]
FIRST	.332	.027	12.33	<.001	[.279,.385]
Tenacity	−.026	.047	−0.54	.586	[−.118,.067]
Strength	−.279	.049	−5.65	<.001	[−.376, −.182]
Optimism	.019	.034	0.57	.567	[−.047,.085]
FIRST × Tenacity	−.045	.047	−0.96	.335	[−.137,.047]
FIRST × Strength	−.101	.043	−2.33	.020	[−.186, −.016]
FIRST × Optimism	.001	.030	0.04	.969	[−.059,.061]

Main-effects model: R^2^ = .327. Full interaction model: R^2^ = .355, adjusted R^2^ = .351. Addition of the three FIRST × resilience-dimension interaction terms: ΔR^2^ = .028, ΔF(3, 1114) = 16.29, p <.001.

All continuous variables, including insomnia symptoms, were standardized as z scores before model estimation. CI, confidence interval; FIRST, Ford Insomnia Response to Stress Test.

The joint interaction model explained 35.5% of the variance in insomnia symptoms (R^2^ = .355, adjusted R^2^ = .351). Relative to the main-effects model (R^2^ = .327), adding the three interaction terms increased the explained variance by 2.8% (ΔR^2^ = .028), ΔF(3, 1114) = 16.29, p <.001. VIFs ranged from 1.26 to 5.64, and tolerance values ranged from.177 to.797. The highest VIF was observed for the FIRST × Strength term (VIF = 5.64, tolerance = .177), although no tolerance value was below.10, indicating no severe multicollinearity.

Additional sensitivity analyses did not consistently support the FIRST × Strength interaction, indicating that the finding was model-dependent (**Supplementary Table 4**). Nevertheless, FIRST × Strength was the only interaction meeting the nominal scale-level selection criterion in the joint OLS model. Strength was therefore carried forward as the moderator in the subsequent exploratory network analysis. Given the mixed sensitivity results, the network analysis was interpreted as a localization of a candidate pattern rather than as independent confirmation of a robust Strength-specific moderation effect.

### 3.3 Moderated network analysis

The results of variable selection using hierarchical BIC-LASSO are presented in **Supplementary Table 1**. Point estimates and 95% confidence intervals from the selected nodewise models and interaction terms are shown in **Supplementary Figures 1 and S2**, respectively. The nodewise adjacency matrix and interaction-term matrix are provided in **Supplementary Tables 2 and S3**.

**Figure 2:**
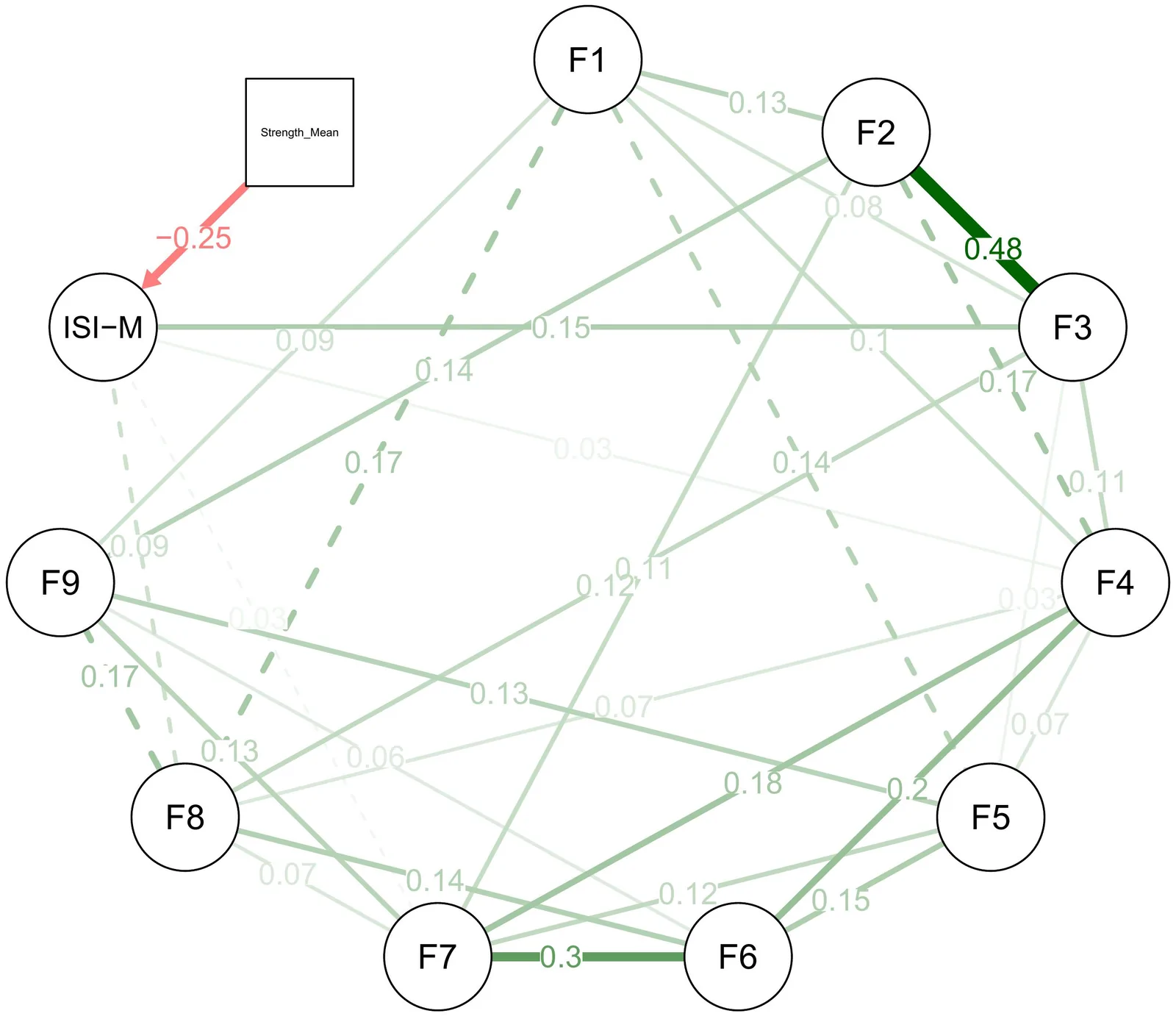
Visualization of the moderated network model with strength as the moderator. The square node represents strength, and the circular nodes represent the ISI composite and the nine FIRST items. Solid lines indicate main (non-interaction) associations between nodes, whereas dashed lines indicate moderation effects by strength retained under the OR rule across nodewise regressions. Green edges denote positive associations, and red edges denote negative associations. Edge thickness is proportional to the absolute value of the estimated coefficients.

As shown in the main-effect network (**Figure 2**), Strength showed a negative main-effect coefficient in the nodewise model predicting the ISI composite (estimate = −.25). The ISI composite was conditionally associated with several FIRST items, with the largest cross-scale association involving sleep reactivity after experiencing a stressful event in the evening (FIRST_3; edge weight = .15); the remaining FIRST–ISI associations were comparatively weaker.

Turning to moderation, the BIC-LASSO selection stage identified 15 candidate directional interaction terms. Following nominal thresholding and application of the OR rule, two retained moderation effects involved the ISI composite: FIRST_7 × Strength (estimate = −.104, SE = .049, 95% CI [−.200, −.009], *p* = .033) and FIRST_8 × Strength (estimate = −.084, SE = .039, 95% CI [−.161, −.008], *p* = .030).

Expected influence (38) was further examined to identify the most central nodes in the main-effect network. As shown in **Supplementary Figure 3**, FIRST_2 had the highest expected influence, followed by FIRST_3, FIRST_4, and FIRST_7, whereas ISI_Mean showed the lowest expected influence. This indicates that several FIRST components occupied more central positions than overall insomnia severity in the estimated network.

**Figure 3:**
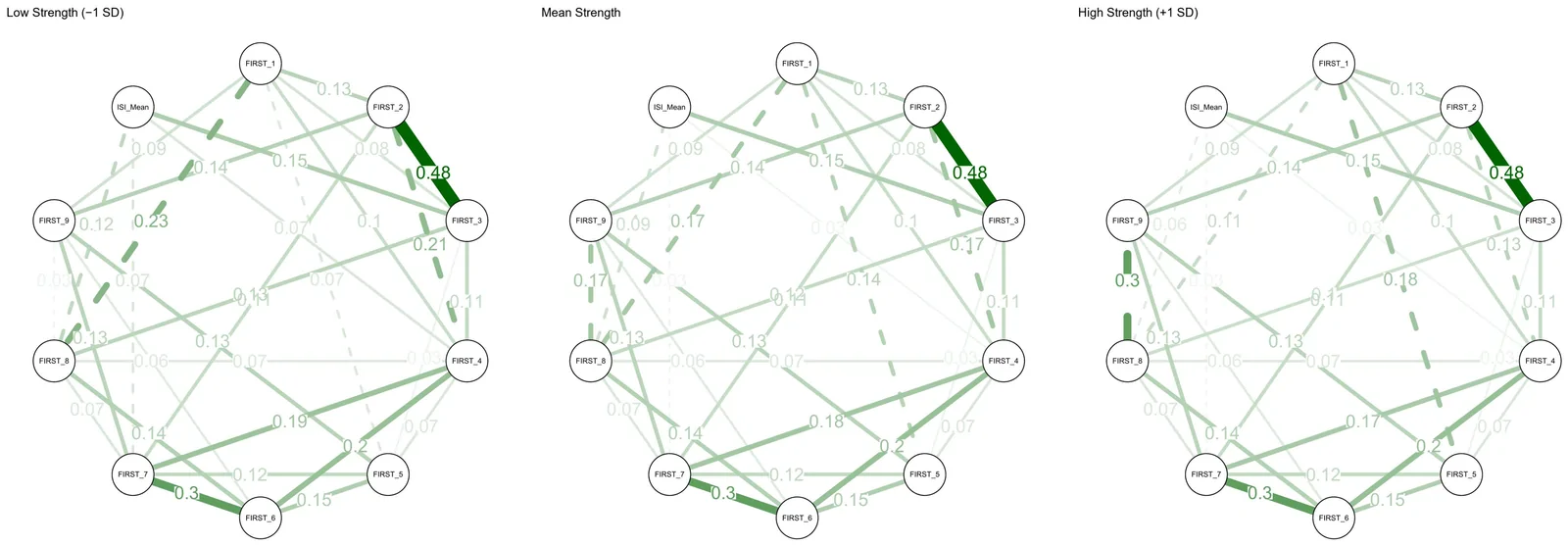
Conditional network models at low (−1 SD), mean, and high (+1 SD) levels of strength. Edge thickness and color represent the magnitude and direction of the conditional associations between nodes (green = positive; red = negative). Dashed lines indicate associations moderated by strength according to the OR rule.

**Figure 4:**
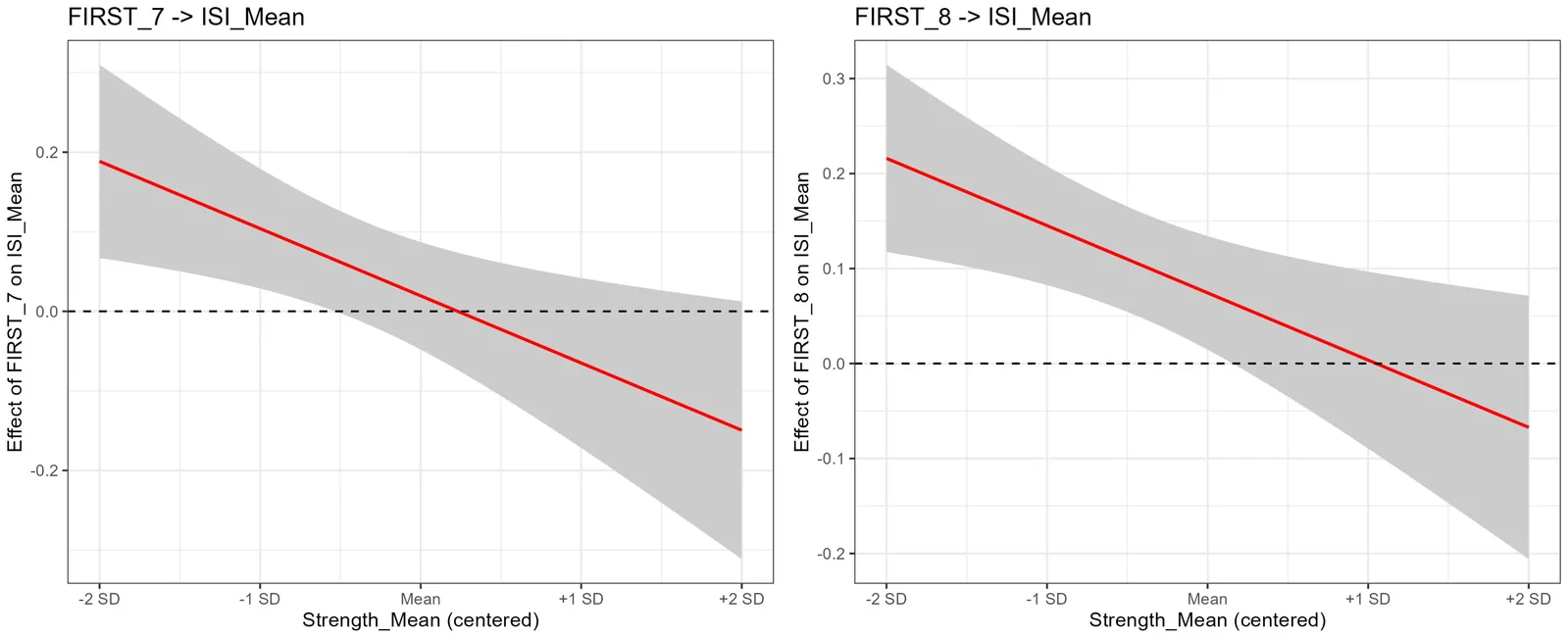
Plots of conditional marginal effects. The left panel shows the conditional marginal effect of FIRST_7 x Strength on the ISI composite, and the right panel shows the conditional marginal effect of FIRST_8 x Strength on the ISI composite. Red lines indicate the estimated conditional marginal effects, gray shaded areas indicate 95% confidence intervals, and the horizontal dashed line indicates zero.

To further examine the moderating role of Strength, conditional networks were estimated at low (-1 SD), mean, and high (+1 SD) levels of Strength (**Figure 3**). The overall network configuration remained broadly similar across the three levels; however, the associations of FIRST_7 and FIRST_8 with ISI_Mean varied as a function of Strength. Specifically, these associations were stronger and positive at lower levels of Strength, but gradually weakened as Strength increased, approaching zero or becoming slightly negative at higher levels of Strength. To further illustrate these moderated edges, **Figure 4** presents the conditional effect plots for FIRST_7-ISI_Mean and FIRST_8-ISI_Mean across levels of Strength. These plots showed the same pattern, indicating that the associations between these specific sleep reactivity components and overall insomnia severity were weaker at higher levels of Strength.

### 3.4 Network stability

The stability of the network parameters was evaluated using the case-dropping subset bootstrap procedure (28). The case-dropping bootstrap plots are presented in **Supplementary Figure 4**, and the corresponding correlation-stability coefficients are summarized in **Table 2**. For the pairwise component, the CS coefficients were.67 for edge weights and.75 for expected influence. For the aggregate interaction component, the corresponding coefficients were.52 and.44. Thus, the pairwise estimates showed good stability, whereas the expected-influence stability of the aggregate interaction structure was acceptable. These interaction coefficients describe the stability of the overall interaction pattern and should not be interpreted as evidence that individual moderation edges were consistently retained.

**Table 2:** Correlation-stability coefficients for the pairwise and interaction components.

Component	Edge weights	Expected influence
Pairwise	.67	.75
Interactions	.52	.44

Values are correlation-stability coefficients based on 1,000 case-dropping bootstrap resamples. The interaction coefficients summarize the aggregate interaction structure and should not be interpreted as the stability of individual moderation edges.

## 4 Discussion

This study examined the association between sleep reactivity and insomnia symptoms and further investigated the role of different dimensions of psychological resilience in this association. Specifically, we first used a multiple regression model to examine the associations of overall sleep reactivity, the three resilience dimensions, and their interaction terms with insomnia symptoms. We then applied a moderated network model to investigate the links between specific components of sleep reactivity and insomnia symptoms at the level of concrete stressful situations.

### 4.1 The moderating role of strength in the association between sleep reactivity and insomnia severity

The multiple regression results showed that sleep reactivity was significantly and positively associated with insomnia symptoms. This finding is consistent with the stress-diathesis model of insomnia. According to this model, insomnia is not caused solely by stressful events; rather, it develops through the joint contribution of individual vulnerability and stress exposure. Sleep reactivity is a specific manifestation of this vulnerability, reflecting the tendency to experience sleep difficulties after stressful situations (5, 8, 15, 39). Recent longitudinal studies further support this view: individuals with high sleep reactivity are more likely to develop acute insomnia or insomnia symptoms after experiencing life stress, and the degree of coupling between daily stress and sleep disturbance can also predict subsequent insomnia risk (14, 17, 40–42). Thus, the positive association between sleep reactivity and insomnia symptoms observed in this study suggests that stress-related sleep vulnerability may be an important correlate of insomnia symptoms. However, the symptom distribution should be considered when interpreting these associations. The findings therefore primarily reflect variation in insomnia symptoms within a predominantly nonclinical and minimally symptomatic sample rather than associations among individuals with clinically significant insomnia.

In the joint OLS model, Strength was negatively associated with insomnia symptoms, whereas the coefficients for Tenacity and Optimism were not statistically significant. Because the three subscales were substantially intercorrelated, this coefficient pattern should not be interpreted as demonstrating a uniquely distinct role for Strength. Conceptually, however, Strength places greater emphasis on the ability to recover, adapt, and maintain psychological stability following stress (19). Its recovery-related content therefore provides one possible theoretical context for interpreting the observed negative association with insomnia symptoms.

More broadly, research has consistently identified associations between sleep and psychological resilience. A systematic review and meta-analysis showed that better sleep quality and longer sleep duration were associated with higher psychological resilience, with the association involving sleep quality being more robust (20). Recent reviews and empirical studies have similarly reported close links among sleep, stress, resilience, and mental health across adolescent and adult samples (22, 43–47). Together, this literature situates the observed association within broader evidence linking sleep and resilience. Importantly, the relationship between sleep and resilience may be reciprocal: persistent insomnia symptoms may also be associated with poorer stress recovery and lower perceived resilience.

Furthermore, the interaction between sleep reactivity and Strength may be understood in terms of post-stress recovery. Resilience does not imply an absence of stress; rather, it refers to the capacity to adapt and recover following stress (18, 48). From this perspective, lower Strength may be associated with greater persistence of worry, rumination, and emotional arousal, whereas higher Strength may facilitate recovery before these responses extend into the presleep period.

### 4.2 The network relationship between sleep reactivity components and insomnia severity

The network analysis showed that insomnia symptoms were connected with multiple sleep reactivity items, with the most prominent link emerging between insomnia symptoms and sleep reactivity after experiencing a stressful event in the evening. This finding suggests that, across different stress-related contexts, sleep reactivity following evening stress may be most closely associated with insomnia symptoms.

This result may be related to the timing of stressful events. Stressful events that occur in the evening are temporally closer to the sleep period, leaving individuals with relatively less time for emotional recovery, cognitive processing, and physiological de-arousal. Therefore, after experiencing stress in the evening, negative emotion, worry, and physiological arousal may not have sufficiently declined before the individual enters the sleep-preparation period. The cognitive model of insomnia emphasizes that worry, rumination, and negative expectations about the consequences of poor sleep during the presleep period are important mechanisms that maintain insomnia symptoms (49). Research on presleep arousal also indicates that both cognitive and somatic arousal are associated with sleep difficulties, with cognitive arousal potentially playing a more prominent role in the association between stress and sleep quality (50–52). Thus, the strong connection between sleep reactivity after evening stress and insomnia symptoms may reflect the greater likelihood that this type of stress enters presleep cognitive and emotional processing.

The perseverative cognition hypothesis may also help explain this finding. According to this hypothesis, repetitive thinking about stressful events can prolong stress-related physiological activation. Even after the stressor itself has ended, sustained cognitive activation may keep individuals in a heightened arousal state (53). Recent longitudinal evidence further shows that rumination and worry play important roles in the bidirectional relationship between stress and sleep quality: stress can affect subsequent sleep through rumination, and poorer sleep quality may in turn increase rumination and worry (54). Another longitudinal study also found that stress affects sleep quality through pathways involving rumination, emotion-focused coping, and mobile phone dependence (55). Therefore, sleep reactivity after evening stress may be more closely linked to insomnia symptoms because this context is more likely to carry post-stress rumination, worry, and high arousal into the presleep period.

By contrast, the connections between sleep reactivity in other stressful situations and insomnia symptoms were relatively weaker. This does not mean that these situations are unrelated to insomnia symptoms; rather, their associations with insomnia symptoms may be more conditional. For example, stressful events that occur during the daytime may also trigger sleep problems, but individuals usually have more time for emotion regulation, attentional shifting, or problem solving, which may reduce their direct impact on presleep states. Other contexts, such as important meetings, bad news, work-related trouble, interpersonal conflict, or public speaking, may also elicit stress-related sleep difficulties, but whether they are closely connected with insomnia symptoms may depend on whether individuals continue to ruminate, worry, or appraise the event as threatening before sleep.

### 4.3 The moderating role of strength in specific sleep reactivity–insomnia links

The exploratory moderated network localized the candidate Strength-related moderation pattern to associations involving sleep reactivity after interpersonal conflict and public speaking. These two moderated associations share a common feature: both involve social stress. Interpersonal conflict involves relational threat, anger, grievance, or self-justification, whereas public speaking involves social evaluation, performance pressure, and the possibility of negative evaluation. Compared with general event-related stress, social stress is more likely to activate processing related to the self, others’ evaluations, and social relationships. Research on social-evaluative threat indicates that when a stressful situation includes evaluation by others and uncontrollability, individuals are more likely to exhibit stronger stress responses. Public speaking tasks are also commonly used to induce social-evaluative stress (56, 57). For men, these situations may also intersect with social expectations concerning relational standing, competence, and emotional composure. Consistent with this possibility, experimental studies have found heightened cortisol responses in men during socially evaluated public-speaking tasks and greater vagal withdrawal when speaking performance was framed as a masculinity threat (58, 59). Therefore, the associations between sleep reactivity after interpersonal conflict or public speaking and insomnia symptoms may be related to social-evaluative worry, interpersonal rumination, and persistent emotional arousal.

In interpersonal conflict situations, individuals may repeatedly recall the argument, evaluate their own or others’ behavior, and worry about relational consequences. Before or after public speaking, individuals may repeatedly anticipate failure, embarrassment, or negative evaluation. These repetitive cognitive activities share features with worry, rumination, and anticipatory stress. The perseverative cognition hypothesis states that repetitive processing of stressful events may prolong post-stress physiological activation (53). Sleep research also shows that rumination, worry, and repetitive negative thinking are related to presleep cognitive arousal and insomnia symptoms (52, 54). In addition, a systematic review of social relationships and sleep quality showed that low-quality or stressful social relationships are usually associated with poorer sleep (60). Thus, the links between sleep reactivity after social stress and insomnia symptoms may partly arise from persistent cognitive processing triggered by interpersonal and evaluative stress.

Taken together, social-evaluative threat theory helps explain why interpersonal conflict and public speaking may elicit particularly self-relevant stress responses (56), whereas the perseverative cognition hypothesis explains how these responses may continue after the stressor has ended and extend into the presleep period (53). Within this combined framework, Strength may be relevant to whether the resulting cognitive and emotional activation persists or is resolved before sleep.

Lower Strength may be accompanied by stronger appraisals of relational threat, incompetence, or loss of self-worth, which could sustain interpersonal rumination, anticipatory worry, and presleep arousal. Higher Strength, by contrast, may support cognitive reappraisal, emotional stabilization, and disengagement from the stressor before sleep, thereby reducing the carryover of social-evaluative stress into the presleep period (18, 48, 49, 56).

This finding also suggests that the moderating effect of Strength may be context specific. Strength did not clearly moderate all links between sleep reactivity items and insomnia symptoms; rather, its moderation was mainly observed in the social stress contexts of interpersonal conflict and public speaking. One possible explanation is that social stress includes not only general stress responses but also self-evaluation, evaluation by others, and relationship security. Such stress is more likely to trigger repetitive thinking and persistent emotional responses, both of which are closely related to insomnia symptoms (49–51, 53). Therefore, the stress recovery capacity and psychological stability represented by Strength may be more likely to exert buffering effects in highly self-relevant and evaluative stress contexts.

In sum, individuals with different levels of Strength may show different mechanisms linking sleep reactivity and insomnia symptoms in the contexts of interpersonal conflict and public speaking. Among individuals with higher Strength, the associations between these two types of social stress-related sleep reactivity and insomnia symptoms were weaker, which may be related to better stress recovery capacity, emotional stability, and adaptive stress appraisal. In contrast, among individuals with lower Strength, these associations were stronger and may be primarily related to interpersonal rumination, social-evaluative worry, and persistent post-stress arousal. Thus, Strength may not uniformly affect sleep reactivity across all stressful situations but may more clearly influence the links between social stress-related sleep reactivity and insomnia symptoms.

## 5 Limitations

Several limitations should be noted. First, the cross-sectional design precludes conclusions regarding temporal or causal directions among sleep reactivity, psychological resilience, and insomnia symptoms. Although prospective evidence suggests that elevated sleep reactivity may precede insomnia, the present associations may also reflect reverse or reciprocal processes. Existing insomnia symptoms may heighten perceived sleep reactivity or compromise resilience-related recovery, while shared factors such as chronic stress, negative affect, and presleep arousal may contribute to all three constructs. Longitudinal and intensive repeated-measures studies are needed to distinguish these possibilities.

Second, participants were recruited through convenience sampling from local universities and were predominantly young and university educated. Consequently, the findings may not generalize to middle-aged or older men, women, clinical populations, or the broader Chinese population. Because no female comparison group was included, the present study cannot determine whether the observed associations or moderation patterns are specific to men. Future studies should recruit more diverse age, educational, regional, and gender groups and directly examine whether these associations differ by gender.

Third, the low and positively skewed ISI distribution, including a substantial concentration at zero, indicates a possible floor effect. The restricted symptom variability may have influenced the magnitude and stability of the estimated associations, and the findings may not generalize to individuals with moderate-to-severe insomnia or clinically diagnosed insomnia.

Fourth, all variables were assessed using self-report scales, which may be affected by common-method bias, recall bias, and social desirability. Although the FIRST, ISI, and CD-RISC have established reliability and validity, future research could incorporate sleep diaries, actigraphy, polysomnography, or physiological arousal indicators to improve the precision of assessments of post-stress sleep responses and insomnia symptoms.

Finally, ISI_Mean was included in the network as a composite measure of overall insomnia severity rather than modeling the seven ISI items separately. This approach reduced model complexity and avoided interpretive problems arising from inconsistent dimensional structures of the ISI. However, it also means that the present study could not further distinguish whether Strength differentially moderates specific insomnia symptoms, such as difficulty initiating sleep, difficulty maintaining sleep, early-morning awakening, daytime impairment, or sleep-related distress.

## 6 Conclusion

The present study showed that, in this sample of young Chinese male university students, sleep reactivity was positively associated with insomnia symptoms, whereas the Strength dimension of psychological resilience was negatively associated with insomnia symptoms and attenuated the association between sleep reactivity and insomnia symptoms. The moderated network results further indicated that the protective role of Strength did not operate uniformly across all stressful situations but was mainly reflected in the links between insomnia symptoms and sleep reactivity related to interpersonal conflict and public speaking. Overall, Strength may represent a capacity for post-stress recovery and psychological stability that helps reduce the coupling between social stress-related sleep vulnerability and insomnia symptoms. Accordingly, future research on stress-related sleep vulnerability among young men may benefit from considering post-stress recovery capacity and adaptive psychological resources alongside sleep reactivity.

## Data Availability

The original contributions presented in the study are included in the article/**Supplementary Material**. Further inquiries can be directed to the corresponding authors.
