# Supplementary material for: The protective role of resilience-related strength in the association between sleep reactivity and insomnia symptoms among young Chinese male university students: a moderated network approach
Source: Front Psychiatry. 2026 Aug 27;17:1905193. doi: 10.3389/fpsyt.2026.1905193 (PMC13558147; doi:10.3389/fpsyt.2026.1905193)
Supplement: Supplementary file 1 [file Supplementaryfile1.docx]

**Supplementary Material: English Item Wording**

# **Ford Insomnia Response to Stress Test (FIRST)**

Instruction: When you experience the following situations, how likely are you to have sleep difficulties? Please choose one response for each situation, even if you have not recently experienced the situation.

Response options: 1 = Not likely, 2 = Slightly likely, 3 = Moderately likely, 4 = Very likely.

1. Before an important meeting the next day.

2. After a stressful experience during the day.

3. After a stressful experience in the evening.

4. After getting bad news during the day.

5. After watching a frightening movie or television show.

6. After having a bad day at work.

7. After an argument.

8. Before having to speak in public.

9. Before going on vacation the next day.

# **Insomnia Severity Index (ISI)**

Instruction: For each item, please select the number that best describes your current insomnia problem, for example, during the past two weeks.

1. Please rate the current severity of your insomnia problem: difficulty falling asleep. Response options: 0 = None, 1 = Mild, 2 = Moderate, 3 = Severe, 4 = Very severe.

2. Please rate the current severity of your insomnia problem: difficulty staying asleep. Response options: 0 = None, 1 = Mild, 2 = Moderate, 3 = Severe, 4 = Very severe.

3. Please rate the current severity of your insomnia problem: waking up too early. Response options: 0 = None, 1 = Mild, 2 = Moderate, 3 = Severe, 4 = Very severe.

4. How satisfied or dissatisfied are you with your current sleep pattern? Response options: 0 = Very satisfied, 1 = Satisfied, 2 = Neutral, 3 = Dissatisfied, 4 = Very dissatisfied.

5. To what extent do you consider your sleep problem to interfere with your daily functioning, such as daytime fatigue, ability to function at work or in daily activities, concentration, memory, or mood? Response options: 0 = Not at all interfering, 1 = A little, 2 = Somewhat, 3 = Much, 4 = Very much interfering.

6. How noticeable to others do you think your sleep problem is in terms of impairing the quality of your life? Response options: 0 = Not at all noticeable, 1 = A little, 2 = Somewhat, 3 = Much, 4 = Very much noticeable.

7. How worried or distressed are you about your current sleep problem? Response options: 0 = Not at all, 1 = A little, 2 = Somewhat, 3 = Much, 4 = Very much.

# **Connor-Davidson Resilience Scale (CD-RISC)**

Instruction: Please indicate how much each statement applies to you over the past month.

Response options: 0 = Not true at all, 1 = Rarely true, 2 = Sometimes true, 3 = Often true, 4 = True nearly all of the time.

1. I am able to adapt to change.

2. I have close and secure relationships.

3. Sometimes fate or God can help.

4. I can deal with whatever comes.

5. Past success gives me confidence in facing new challenges.

6. I can see the humorous side of things.

7. Coping with stress can strengthen me.

8. I tend to bounce back after illness or hardship.

9. Things happen for a reason.

10. I give my best effort no matter what the outcome may be.

11. I am able to meet my goals.

12. I do not give up easily when things look hopeless.

13. I know where to turn for help.

14. Under pressure, I can focus and think clearly.

15. I prefer to take the lead in problem solving.

16. I am not easily discouraged by failure.

17. I think of myself as a strong person.

18. I can make unusual or difficult decisions.

19. I can handle unpleasant feelings.

20. I have to act on a hunch.

21. I have a strong sense of purpose.

22. I feel in control of my life.

23. I like challenges.

24. I work hard to attain my goals.

25. I take pride in my achievements.
