# Supplementary material for: The protective role of resilience-related strength in the association between sleep reactivity and insomnia symptoms among young Chinese male university students: a moderated network approach
Source: Front Psychiatry. 2026 Aug 27;17:1905193. doi: 10.3389/fpsyt.2026.1905193 (PMC13558147; doi:10.3389/fpsyt.2026.1905193)
Supplement: Supplementary file 2 [file Supplementaryfile2.docx]

**Supplementary Material**

**Supplementary Methods**

**Regression robustness analyses**

The stability of the FIRST × Strength interaction was examined using HC3 heteroscedasticity-consistent standard errors, nonparametric bootstrap confidence intervals based on 2,000 resamples, Huber robust regression, and a negative-binomial model for the ISI total score. Influential observations were identified using Cook's distance with a threshold of 4/N, after which the joint interaction model was refitted with the identified observations excluded. Complete results are reported in Supplementary Table S4.

**Covariate-adjusted sensitivity analysis**

To examine potential demographic and behavioral confounding, the joint interaction model was additionally adjusted for age, education, smoking, and alcohol use. Age was entered as a continuous covariate. Education was dichotomized as high school or vocational education or below versus university education or above; smoking and alcohol use were entered as binary variables (no vs. yes). The standardized ISI, FIRST, and three CD-RISC subscale scores, together with the three FIRST-by-resilience interaction terms, were specified as in the primary regression model. Both conventional ordinary least squares (OLS) and HC3 heteroscedasticity-consistent inference were examined. All 1,122 participants had complete data for these covariates. Complete results are reported in Supplementary Table S5.

**Moderated-network estimation and stability**

The primary Gaussian moderated network included the nine FIRST items as separate nodes, the mean of the seven ISI items as a composite insomnia-severity node, and Strength as the moderator. The ISI mean is a linear rescaling of the conventional ISI total score and was used to represent overall insomnia severity while limiting model complexity; accordingly, the primary network was not interpreted at the level of individual insomnia symptoms.

Variable and interaction selection was performed using hierarchical LASSO implemented with varSelect in the modnets package. The regularization parameter was selected using BIC, and the resulting selection object was passed to fitNetwork for nodewise estimation. Variables were internally mean-centered but were not scaled to unit variance (center = TRUE, scale = FALSE, std = FALSE). The random seed was 999. For visualization, a nominal p < .05 threshold and the OR rule were applied, such that an edge was retained when at least one of the corresponding nodewise regressions met the threshold. The OR rule was used because the network analysis was intended as an exploratory localization analysis. No formal multiple-testing correction was applied to the LASSO selection stage.

Case-dropping stability was assessed with 1,000 resamples using the selected model held fixed during refitting. Correlation-stability coefficients were calculated for the pairwise and overall interaction coefficient structures and for expected influence. The interaction coefficients describe stability of the aggregate interaction pattern and do not establish that any individual moderation edge was consistently selected.

**Supplementary Table S1.** Hierarchical BIC-LASSO variable-selection results for the primary moderated network (N = 1,122).

| **label** | **FIRST_1** | **FIRST_2** | **FIRST_3** | **FIRST_4** | **FIRST_5** | **FIRST_6** | **FIRST_7** | **FIRST_8** | **FIRST_9** | **ISI_Mean** |
| --- | --- | --- | --- | --- | --- | --- | --- | --- | --- | --- |
| mods | FIRST_2 | FIRST_1 | FIRST_1 | FIRST_1 | FIRST_1 | FIRST_1 | FIRST_2 | FIRST_1 | FIRST_1 | FIRST_3 |
|  | FIRST_3 | FIRST_3 | FIRST_2 | FIRST_2 | FIRST_3 | FIRST_2 | FIRST_3 | FIRST_2 | FIRST_2 | FIRST_4 |
|  | FIRST_4 | FIRST_4 | FIRST_4 | FIRST_3 | FIRST_4 | FIRST_3 | FIRST_4 | FIRST_3 | FIRST_5 | FIRST_6 |
|  | FIRST_5 | FIRST_6 | FIRST_5 | FIRST_5 | FIRST_6 | FIRST_4 | FIRST_5 | FIRST_4 | FIRST_6 | FIRST_7 |
|  | FIRST_6 | FIRST_7 | FIRST_6 | FIRST_6 | FIRST_7 | FIRST_5 | FIRST_6 | FIRST_5 | FIRST_7 | FIRST_8 |
|  | FIRST_8 | FIRST_8 | FIRST_7 | FIRST_7 | FIRST_8 | FIRST_7 | FIRST_8 | FIRST_6 | FIRST_8 | FIRST_9 |
|  | FIRST_9 | FIRST_9 | FIRST_8 | FIRST_8 | FIRST_9 | FIRST_8 | FIRST_9 | FIRST_7 | Strength_Mean | Strength_Mean |
|  | Strength_Mean | Strength_Mean | ISI_Mean | ISI_Mean |  | FIRST_9 | ISI_Mean | FIRST_9 |  |  |
|  |  |  |  | Strength_Mean |  | ISI_Mean |  | ISI_Mean |  |  |
|  |  |  |  |  |  |  |  | Strength_Mean |  |  |
| ints | FIRST_5:Strength_Mean | FIRST_4:Strength_Mean |  | FIRST_2:Strength_Mean |  |  |  | FIRST_1:Strength_Mean | FIRST_8:Strength_Mean | FIRST_3:Strength_Mean |
|  | FIRST_8:Strength_Mean |  |  | FIRST_7:Strength_Mean |  |  |  | FIRST_3:Strength_Mean |  | FIRST_4:Strength_Mean |
|  |  |  |  |  |  |  |  | FIRST_5:Strength_Mean |  | FIRST_7:Strength_Mean |
|  |  |  |  |  |  |  |  | FIRST_9:Strength_Mean |  | FIRST_8:Strength_Mean |
|  |  |  |  |  |  |  |  |  |  | FIRST_9:Strength_Mean |

*Note.* Columns denote nodewise outcomes. The rows labelled mods and ints list the selected lower-order and interaction terms, respectively. BIC = Bayesian information criterion; LASSO = least absolute shrinkage and selection operator.

**Supplementary Table S2.** Pairwise adjacency matrix for the primary FIRST-ISI moderated network (OR rule; threshold = TRUE; N = 1,122).

|  | **FIRST_1** | **FIRST_2** | **FIRST_3** | **FIRST_4** | **FIRST_5** | **FIRST_6** | **FIRST_7** | **FIRST_8** | **FIRST_9** | **ISI_Mean** |
| --- | --- | --- | --- | --- | --- | --- | --- | --- | --- | --- |
| FIRST_1 | 0 | .135 | .082 | .104 | .138 | 0 | 0 | .169 | .091 | 0 |
| FIRST_2 | .135 | 0 | .482 | .171 | 0 | 0 | .109 | 0 | .136 | 0 |
| FIRST_3 | .082 | .482 | 0 | .114 | .034 | 0 | 0 | .116 | 0 | .152 |
| FIRST_4 | .104 | .171 | .114 | 0 | .071 | .197 | .175 | .069 | 0 | .033 |
| FIRST_5 | .138 | 0 | .034 | .071 | 0 | .152 | .117 | 0 | .134 | 0 |
| FIRST_6 | 0 | 0 | 0 | .197 | .152 | 0 | .303 | .145 | .061 | 0 |
| FIRST_7 | 0 | .109 | 0 | .175 | .117 | .303 | 0 | .072 | .131 | .027 |
| FIRST_8 | .169 | 0 | .116 | .069 | 0 | .145 | .072 | 0 | .175 | .094 |
| FIRST_9 | .091 | .136 | 0 | 0 | .134 | .061 | .131 | .175 | 0 | 0 |
| ISI_Mean | 0 | 0 | .152 | .033 | 0 | 0 | .027 | .094 | 0 | 0 |

*Note.* Matrix entries are conditional pairwise coefficients. A value of zero indicates that the corresponding edge was not retained.

**Supplementary Table S3.** Directional interaction-term matrix for the primary FIRST-ISI moderated network with Strength as moderator (OR rule; threshold = TRUE; N = 1,122).

|  | **FIRST_1** | **FIRST_2** | **FIRST_3** | **FIRST_4** | **FIRST_5** | **FIRST_6** | **FIRST_7** | **FIRST_8** | **FIRST_9** | **ISI_Mean** |
| --- | --- | --- | --- | --- | --- | --- | --- | --- | --- | --- |
| FIRST_1:Strength_Mean | 0 | 0 | 0 | 0 | 0 | 0 | 0 | -.096 | 0 | 0 |
| FIRST_2:Strength_Mean | 0 | 0 | 0 | 0 | 0 | 0 | 0 | 0 | 0 | 0 |
| FIRST_3:Strength_Mean | 0 | 0 | 0 | 0 | 0 | 0 | 0 | 0 | 0 | 0 |
| FIRST_4:Strength_Mean | 0 | -.071 | 0 | 0 | 0 | 0 | 0 | 0 | 0 | 0 |
| FIRST_5:Strength_Mean | .133 | 0 | 0 | 0 | 0 | 0 | 0 | 0 | 0 | 0 |
| FIRST_6:Strength_Mean | 0 | 0 | 0 | 0 | 0 | 0 | 0 | 0 | 0 | 0 |
| FIRST_7:Strength_Mean | 0 | 0 | 0 | 0 | 0 | 0 | 0 | 0 | 0 | -.104 |
| FIRST_8:Strength_Mean | -.101 | 0 | 0 | 0 | 0 | 0 | 0 | 0 | .157 | -.084 |
| FIRST_9:Strength_Mean | 0 | 0 | 0 | 0 | 0 | 0 | 0 | .244 | 0 | 0 |
| ISI_Mean:Strength_Mean | 0 | 0 | 0 | 0 | 0 | 0 | 0 | 0 | 0 | 0 |

*Note.* Rows denote predictor-by-Strength interaction terms and columns denote nodewise outcomes. A value of zero indicates that the directional coefficient did not meet the retention rule.

**Supplementary Table S4.** Sensitivity analyses for the FIRST × Strength interaction.

| **Analysis** | **N** | **Estimate** | **SE** | **p** | **95% CI** |
| --- | --- | --- | --- | --- | --- |
| Primary joint OLS | 1,122 | -.101 | .043 | .020 | [-.186, -.016] |
| HC3 standard errors | 1,122 | -.101 | .069 | .141 | [-.236, .034] |
| Bootstrap percentile CI (2,000) | 1,122 | -.101 | - | - | [-.246, .016] |
| Bootstrap BCa CI (2,000) | 1,122 | -.101 | - | - | [-.242, .017] |
| Huber robust regression | 1,122 | -.118 | .028 | - | [-.173, -.063] |
| Negative-binomial ISI model | 1,122 | .086 | .065 | .183 | [-.041, .212] |
| Influential cases excluded, OLS | 1,040 | -.120 | .049 | .014 | [-.217, -.024] |
| Influential cases excluded, HC3 | 1,040 | -.120 | .046 | .008 | [-.210, -.031] |

*Note.* Eighty-two observations exceeded Cook's D > 4/N and were excluded from the influence sensitivity models. The negative-binomial coefficient is reported on the log-count scale and is not directly comparable with the standardized linear-model coefficients. Dashes indicate statistics not reported for that procedure. BCa = bias-corrected and accelerated; HC3 = heteroscedasticity-consistent standard errors.

**Supplementary Table S5. Covariate-adjusted sensitivity analysis of the interactions between sleep reactivity and resilience dimensions (N = 1,122).**

| **Inference** | **Interaction** | **Estimate** | **SE** | **p** | **95% CI** |
| --- | --- | --- | --- | --- | --- |
| OLS | FIRST × Tenacity | -.047 | .047 | .320 | [-.139, .045] |
| OLS | FIRST × Strength | -.097 | .043 | .025 | [-.182, -.012] |
| OLS | FIRST × Optimism | -.001 | .031 | .964 | [-.061, .059] |
| HC3 | FIRST × Tenacity | -.047 | .070 | .507 | [-.185, .091] |
| HC3 | FIRST × Strength | -.097 | .069 | .157 | [-.232, .038] |
| HC3 | FIRST × Optimism | -.001 | .061 | .982 | [-.122, .119] |

*Note. All models controlled for age, education, smoking, and alcohol use. Age was entered as a continuous covariate; education was coded as high school or vocational education or below versus university education or above; and smoking and alcohol use were coded as no versus yes. The covariate-adjusted model yielded R² = .357 and adjusted R² = .350, compared with R² = .355 and adjusted R² = .351 for the unadjusted joint model. Estimate denotes the standardized coefficient for each interaction term. HC3 = heteroscedasticity-consistent standard errors; OLS = ordinary least squares.*

**Supplementary Figure S1.** Point estimates and 95% confidence intervals from the selected FIRST, ISI, and Strength nodewise models (N = 1,122).

**Supplementary Figure S2.** Point estimates and 95% confidence intervals for the interaction terms selected by hierarchical BIC-LASSO (N = 1,122).

**Supplementary Figure S3.** Expected-influence values for the FIRST items and ISI composite in the pairwise network (N = 1,122). Expected influence represents the signed sum of all pairwise edge weights connected to a node and does not imply causal importance.

**Supplementary Figure S4.** Case-dropping stability plots based on 1,000 resamples. Pairwise CS coefficients were .67 for edges, .75 for strength, and .75 for expected influence; interaction CS coefficients were .52, .52, and .44, respectively. Interaction coefficients describe aggregate pattern stability rather than stability of individual moderation edges.
